# A piecewise sine waveguide for terahertz traveling wave tube

**DOI:** 10.1038/s41598-022-14587-y

**Published:** 2022-06-21

**Authors:** Luqi Zhang, Yi Jiang, Wenqiang Lei, Peng Hu, Jun Guo, Rui Song, Xianfeng Tang, Guowu Ma, Hongbin Chen, Yanyu Wei

**Affiliations:** 1 Institute of Applied Electronics, China Academy of Engineering Physics, Mianyang, 621900 China; 2 Department of Physics, Southwest Jiao Tong University, Chengdu, 610031 China; 3 School of Electronic Science and Engineering, University of Electronic Science and Technology University of China, Chengdu, 610054 China

**Keywords:** Electronics, photonics and device physics, Electrical and electronic engineering

## Abstract

In this paper, a piecewise sine waveguide (PWSWG) is proposed as the slow-wave structure (SWS) to develop high-power terahertz (THz) traveling wave tubes (TWTs). The PWSWG is an improvement over the rectangular waveguide wherein its two E-planes simultaneously oscillate up and down along the longitudinal direction. The oscillation curve in the H-plane is a piecewise sine curve formed by inserting line segments into the peaks and troughs of the sine curve. The simulation analysis and experimental verification show that the PWSWG offers the advantages of large interaction impedance and excellent electromagnetic transmission performance. Furthermore, the calculation results of beam–wave interaction show that the TWT based on PWSWG SWS can generate a radiated power of 253.1 W at the typical frequency of 220 GHz, corresponding to a gain of 37.04 dB and an interaction efficiency of 6.92%. Compared with the conventional SWG TWTs, the PWSWG TWT has higher interaction efficiency and shorter saturation tube length. In conclusion, the PWSWG proposed in this paper can be considered a suitable SWS for high-power THz radiation sources.

## Introduction

Terahertz (THz) technology has tremendous application prospects in high-speed wireless communication, high-resolution synthetic aperture radar imaging, biomedical diagnosis, and space exploration^1–4^. The study of THz radiation is one of the most important research topics in this field^5^. Benefiting from the energy conversion mechanisms of electron beams and electromagnetic waves^6^, vacuum electronic devices (VEDs) are well suited for generating high-power THz radiation. Of all the THz VEDs, THz traveling wave tubes (TWTs) yield superior performance in terms of bandwidth and power capacity^7^. Therefore, THz TWTs have immense application potential in THz electromagnetic systems.

As the core component of the THz TWT, the slow-wave structure (SWS) directly determines the device performance of this high-power THz radiation source. Many types of SWSs, such as folded waveguide^8,9^, corrugated rectangular waveguide^10,11^, double corrugated rectangular waveguide^12^, and staggered double-gate structure^13,14^, have been proposed for developing high-power radiation sources in the THz band. However, at high operating frequencies, the high transmission loss and strong reflection in these SWSs limit the output power and bandwidth of the THz TWT. The sine waveguide (SWG) SWS, which exhibits excellent transmission performance due to its uniform cross-section and simple energy coupling structures, has been employed for designing THz VEDs^15,16^. However, the longitudinal electrical field intensity of the SWG SWS is very low, resulting in the low interaction impedance offered by this structure^17^. Some modified SWGs, such as the flat-roofed SWG (FRSWG) and the sine-shaped ridge waveguide, have been presented to improve the interaction impedance^18,19^, but they either destroy the uniform cross-section or pose difficulties in machining. For example, some recent studies on FRSWG SWS reported that the relative bandwidth of the THz TWT fails to exceed 10% due to the destruction of the longitudinal uniformity of the SWS^20–22^. Thus, how to achieve a high interaction impedance with excellent electromagnetic transmission performance in the THz SWS is an important issue in the development of high-power THz TWTs.

Different from the existing THz SWSs, a novel THz SWS is proposed in this paper to improve the electric field distribution and avoid the leap point by constructing the piecewise sine boundary of the SWS. A distinctive piecewise SWG (PWSWG), which can be considered a suitable SWS for high-power THz radiation sources, is analyzed for the first time in this paper. This paper is organized as follows. In “Slow‑wave characteristics” section, the slow-wave characteristics of the PWSWG SWS are analyzed. The physical principle of interaction impedance growth is also explained in “Slow‑wave characteristics” section. In “Transmission properties” section, the beam–wave interaction circuit is developed based on the PWSWG SWS, and the transmission parameters are obtained by combining the simulation calculation with the experimental test. The beam–wave interaction performance of the G-band PWSWG TWT is predicted using 3D particle-in-cell (PIC) algorithms in “Beam–wave interaction performance” section. Finally, the summary of this work is given in “Summary” section.

## Slow-wave characteristics

**Figure 1:**
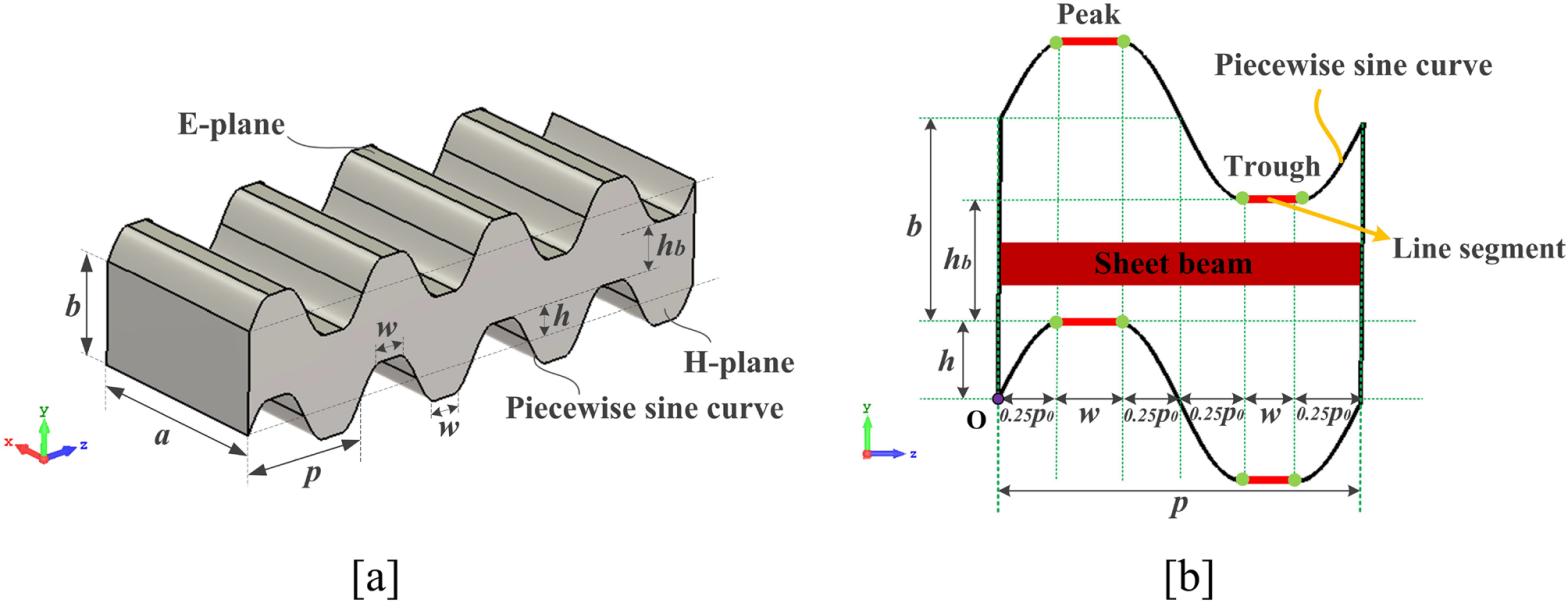
3-D structure model and dimensional parameters: (**a**) perspective view and (**b**) side view.

Figure 1a shows the PSRWG SWS, which is an improvement over the rectangular waveguide, with the wide-side and narrow-side lengths as *a* and *b*, respectively. Its two E-planes simultaneously oscillate up and down along the longitudinal direction with an amplitude *h* and a period *p*. The oscillation amplitude should not exceed half of the narrow-side length of the rectangular waveguide. Under these conditions, the cross-section of the PSRWG SWS can be kept uniform, and a rectangular electron channel with a cross-sectional area of *a* × *h*_*b*_ can be formed. As can be seen from Fig. 1b, the oscillation curve in the H-plane is a piecewise sine curve formed by inserting line segments into the peaks and troughs of the sine curve. The length of the line segment is *w*, and the period of the sine curve is *p*_*0*_.

**Figure 2:**
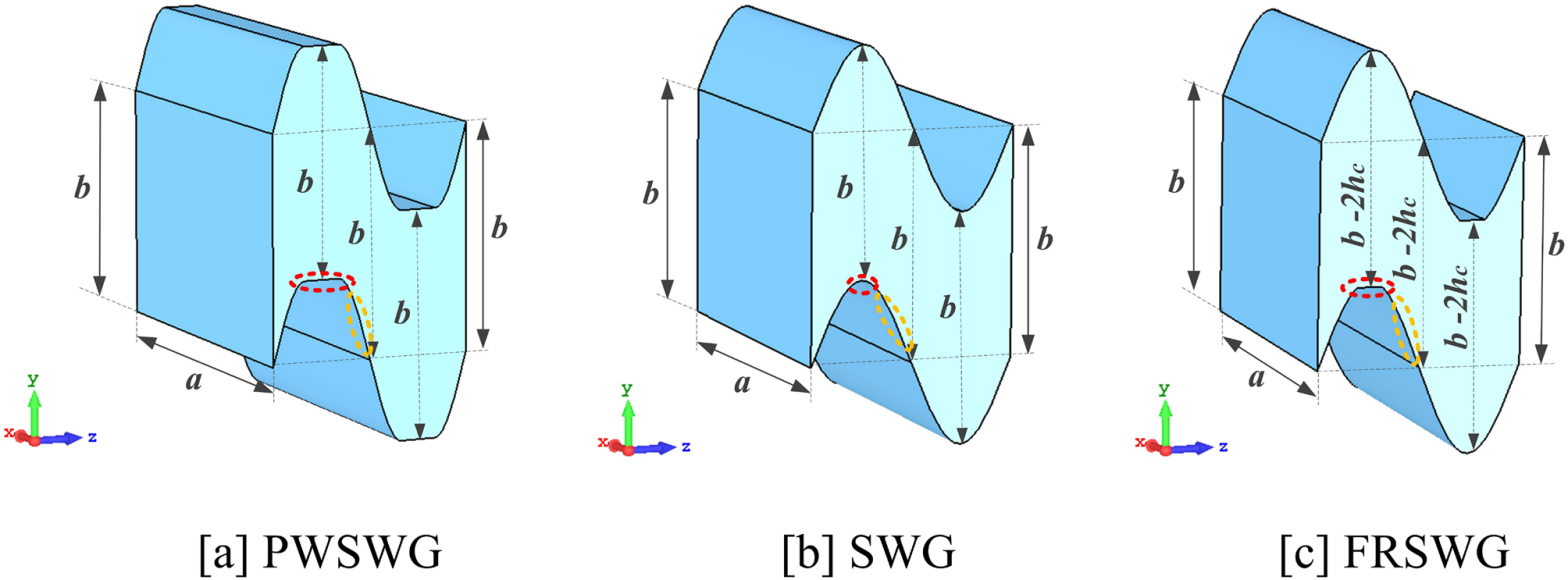
Comparisons of the 3D structure model of the PWSWG, the SWG and the FRSWG.

The PWSWG SWS proposed in this paper is novel in terms of two aspects. First, for the three structures shown in Fig. 2, the longitudinal electric field distribution in the interaction region is primarily determined by the width of the metal gate (marked as a red circle) and the sinusoidal curvature (marked as a yellow circle). In the FRSWG SWS, the width of the metal gate can be changed by varying the flattened height to improve the longitudinal electric field intensity. However, the curvature of the sine curve does not change with changes in the flattened height. In the PWSWG SWS proposed in this paper, the width of the metal gate and the curvature of the sine curve can be changed simultaneously. Thus, an additional adjustable physical parameter is made available to improve the longitudinal electric field distribution to maximize the longitudinal electric field strength.

For this reason, the PWSWG SWS with the same dimensional parameters has a higher interaction impedance than the other two SWSs. Second, in the propagation direction of the electromagnetic wave, the cross-section of the PWSWG SWS is uniform and rectangular (*a* × *b*). In contrast, the FWSWG has a variable cross-section, as shown in Fig. 2c. As a result, the PWSWG SWS offers the advantages of the SWG SWS operating in a wide frequency range, whereas the operating bandwidth of the FRSWG SWS is relatively limited. The PWSWG is a new type of THz SWS with a high interaction impedance and excellent electromagnetic transmission properties.

**Figure 3:**
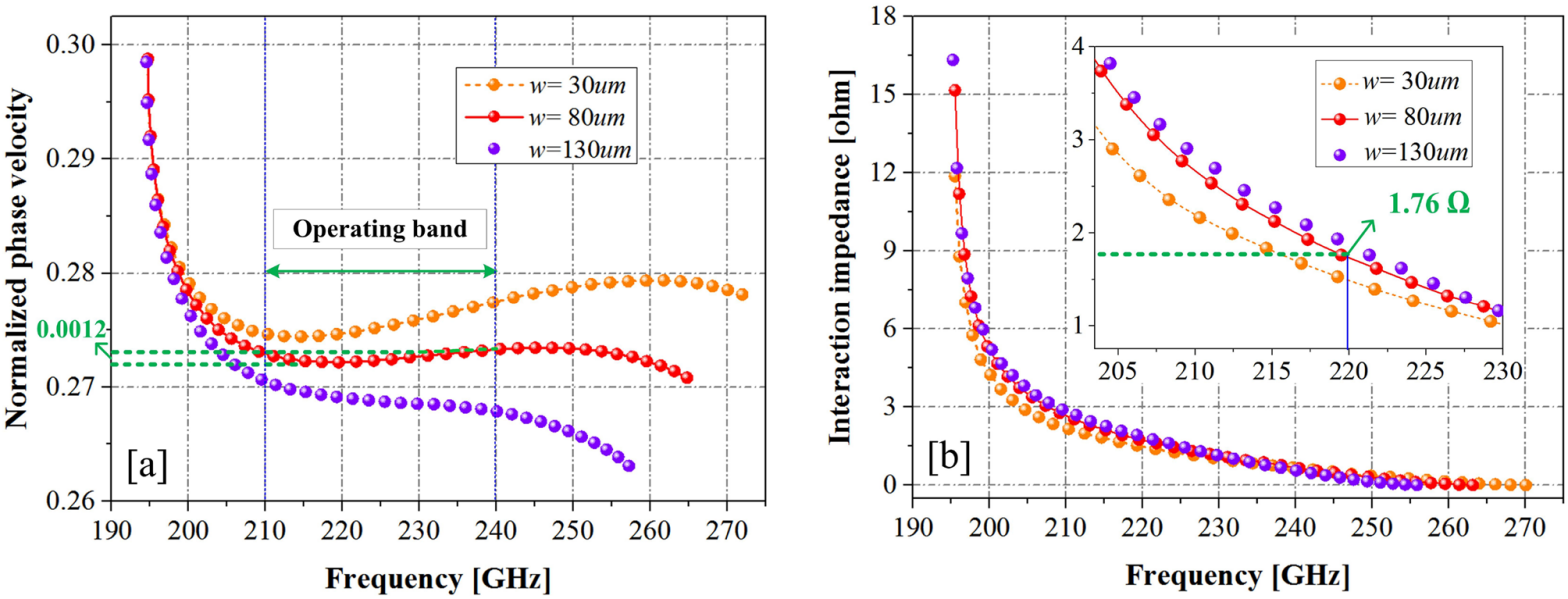
Dispersion properties and interaction impedances (from HFSS) of the PSRWG SWS with different *w.*

The Eigen-mode solver in high-frequency structure simulator 15.0 (HFSS)^23^, a 3D electromagnetic simulation software tool, was used to analyze the slow-wave characteristics of the PWSWG SWS. To realize broad matching in the frequency range of 210–240 GHz, the dimensional parameters of the PWSWG SWS were partially optimized through calculations and selected as follows: *a* = 770 µm, *p* = 460 µm, *h* = 180 µm, and *h*_*b*_ = 140 µm. For the PWSWG SWS, the line-segment length *w* is the key structural parameter that affects its slow-wave characteristics. In addition, the influence of the line-segment length on dispersion properties and interaction impedances was analyzed via simulation, as shown in Fig. 3. The phase velocity (Fig. 3) was normalized to the light wave velocity. As can be seen in Fig. 3a, the normalized phase velocity decreased, and the cold bandwidth became narrow as the line-segment length *w* was increased from 30 to 130 µm. Moreover, it can be seen from Fig. 3b that the interaction impedance values increased gradually with the increase in the line-segment length *w*. The optimization process is aimed to maximize the interaction impedance under the condition that the dispersion curve in the operating band (210–240 GHz) is relatively flat. Thus, the dispersion flatness and interaction impedance are the main factors considered to reach a compromise. The dispersion curves were obtained by changing the value of the line-segment length *w*, as given in Fig. 3. Additionally, the normalized phase velocity fluctuation was set within the working frequency band not to exceed 0.0012 so that the corresponding optimal synchronization voltage fluctuation did not exceed 200 V. Under this condition, the maximum interaction impedance of 1.76 Ω was obtained at 220 GHz, and the corresponding segment length was optimized to 80 µm.

**Figure 4:**
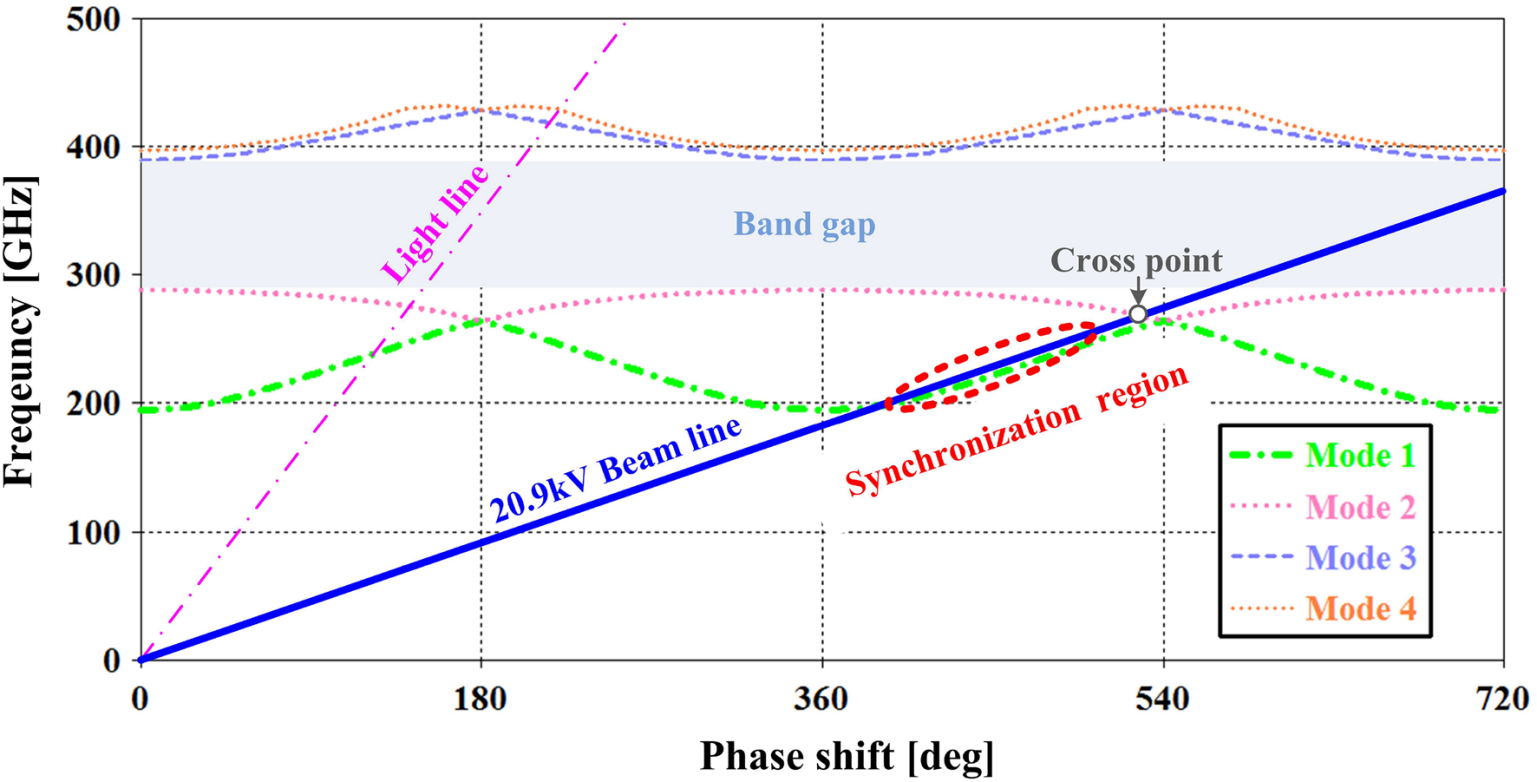
The Brillouin curve (from HFSS): the dash dot red line is the dispersion curve of the fundamental mode in the PWSWG SWS, the solid blue line is the electron-beam curve with a voltage 20.9 kV.

The Brillouin curve of the PWSWG SWS was plotted using the structural parameters obtained from the above optimization (Fig. 4). To ensure the best synchronization at 220 GHz, the electron beam voltage was selected as 20.9 kV. From Fig. 4, it can be seen that the PWSWG SWS has a main competitive mode (Mode 2) that can cause back-wave oscillation. Although the interaction impedance at the cross point is approximately 0.15 Ω, this risk of oscillation must be considered during the actual device design^24^.

**Figure 5:**
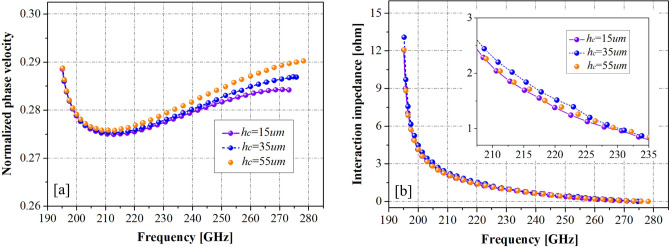
Dispersion properties and interaction impedances of the FRSWG SWS with different *h*_*c*_*.*

The slow-wave characteristics of the PWSWG SWS, SWG SWS, and FRSWG SWS were compared via simulation. The three SWSs exhibited similar structural parameters (Table 1). For a fair comparison, most dimensional parameters of the FRSWG SWS were the same as those of the PWSWG SWS. The key structural parameter *h*_*c*_ for the FRSWG SWS was optimized^25^ (Fig. 5). It can be seen in Fig. 5b that the interaction impedance of the FRSWG SWS is the highest when the flattened height *h*_*c*_ is 35 µm.

**Table 1:** Structural parameters of the PWSWG SWS, the SWG SWS and the FRSWG SWS.

Parameter	PWSWG SWS (µm)	SWG SWS (µm)	FRSWG SWS (µm)
Wide side length *a*	770	770	770
Oscillating amplitude *h*	180	180	180
Oscillating period *p*	460	460	460
Beam tunnel height *h*_*b*_	140	140	140
Line segment length *d*	80	–	–
Flattened height *h*_*c*_	–	–	35

**Figure 6:**
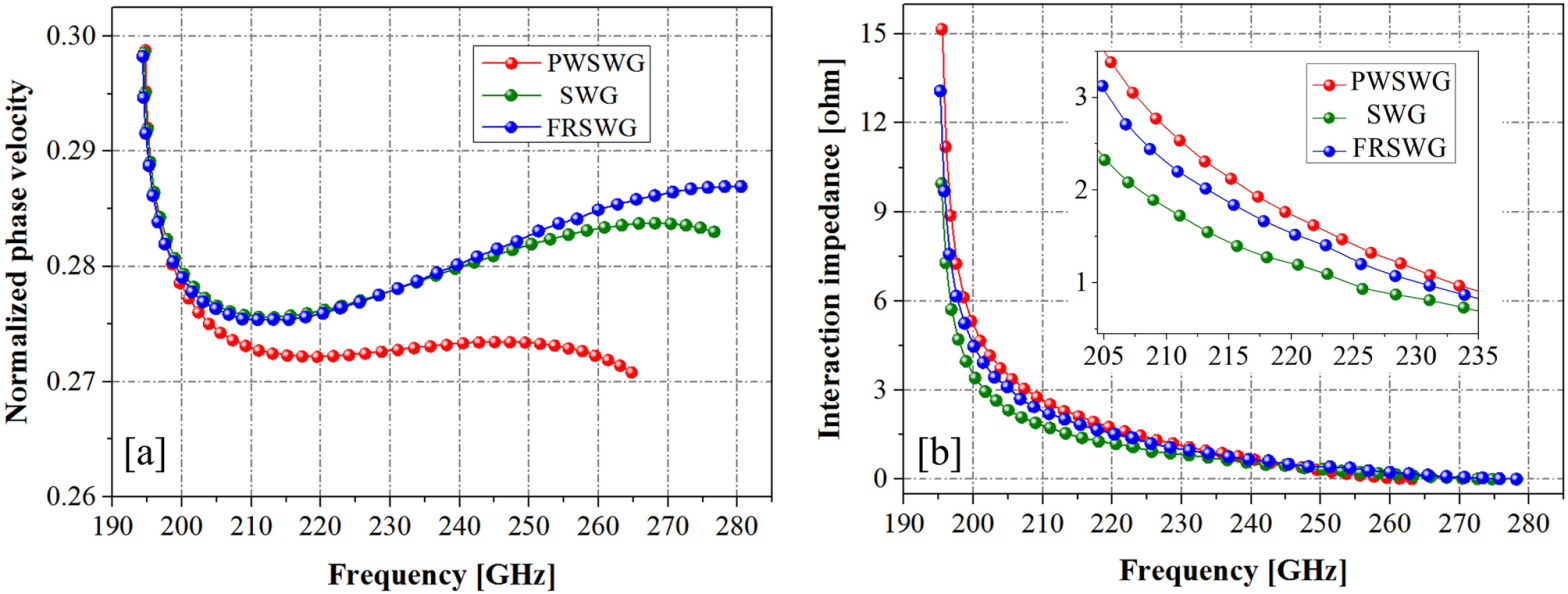
Comparisons of slow wave characteristics between the PWSWG SWS, the SWG SWS and the FRSWG SWS.

Figure 6 shows that the PWSWG SWS has the flattest dispersion curve and the lowest normalized phase velocity among the three SWSs. From the synchronization conditions, it can be inferred that the PWSWG SWS has wider bandwidth and lower synchronization voltage^24^. Furthermore, the interaction impedance of the PWSWG SWS is considerably higher than that of the other two SWSs in the frequency range of 210–240 GHz. In particular, the interaction impedance of the PWSWG SWS at 220 GHz is approximately 46.7% and 16.6% higher than that of the SWG SWS and FRSWG SWS, respectively.

The mechanism of interaction impedance growth was studied using interaction impedance *K*_*c*_, which is expressed using Eq. (1). where *P*_*w*_ is the transmission power flow on the axis, and *E*_*zn*_ and β_*n*_ are, respectively, the amplitude of the longitudinal electric field and the phase constant of the *n*-th spatial harmonic. For the three SWSs, the TWT operates on the first positive space harmonic (*n* = 1).

1 $$K_{c} = \frac{{|E_{zn} |^{2} }}{{2\beta_{n}^{2} P_{w} }}.$$

**Figure 7:**
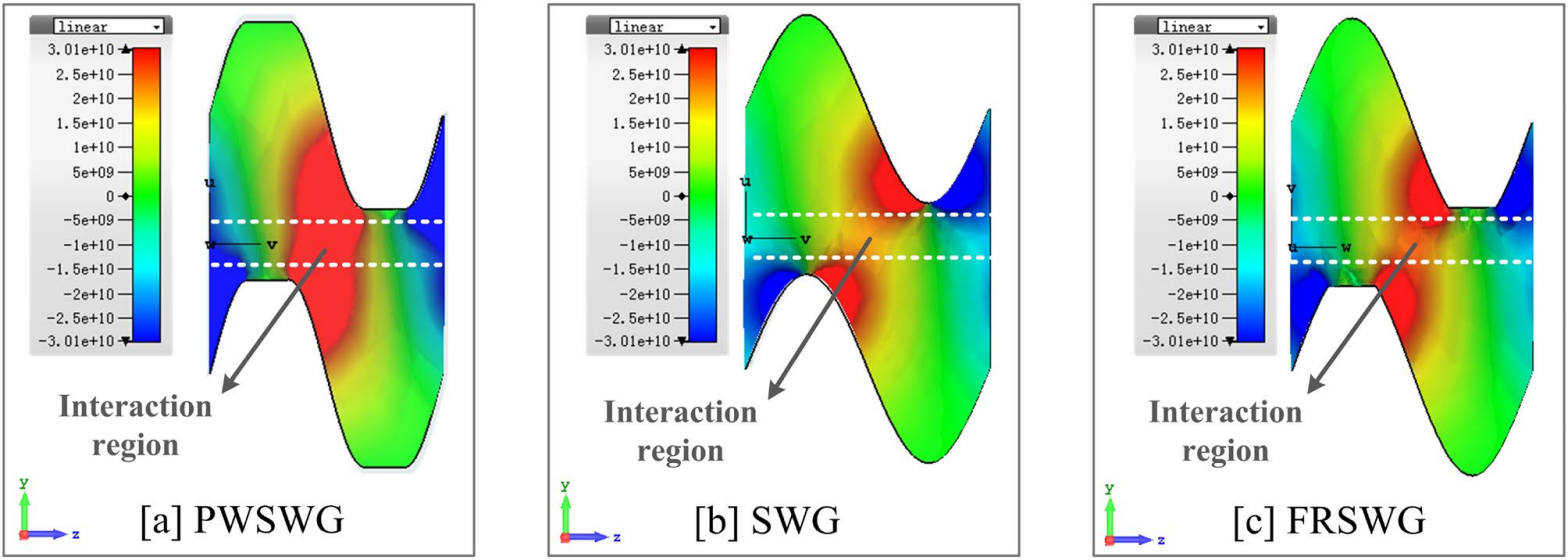
Longitudinal electrical field of the SSRWG SWS, the SWG SWS and the FRSWG SWS at 220 GHz.

The longitudinal electrical field *E*_*z*_ of the three SWSs at the typical frequency of 220 GHz was calculated (Fig. 7). For the PWSWG SWS, the longitudinal electric field distribution was improved by adjusting the line-segment length. The longitudinal electrical field amplitude of the PWSWG SWS was much greater than that of the other two SWSs in the beam–wave interaction region. This is the most important factor in the improvement of the interaction impedance. The interaction impedance growth in the SWS indicates an increase in the coupling strength between the electromagnetic wave and the electron beam. As a result, we can speculate that the TWT based on the PWSWG SWS may have higher interaction efficiency and better amplification performance.

## Transmission properties

**Figure 8:**
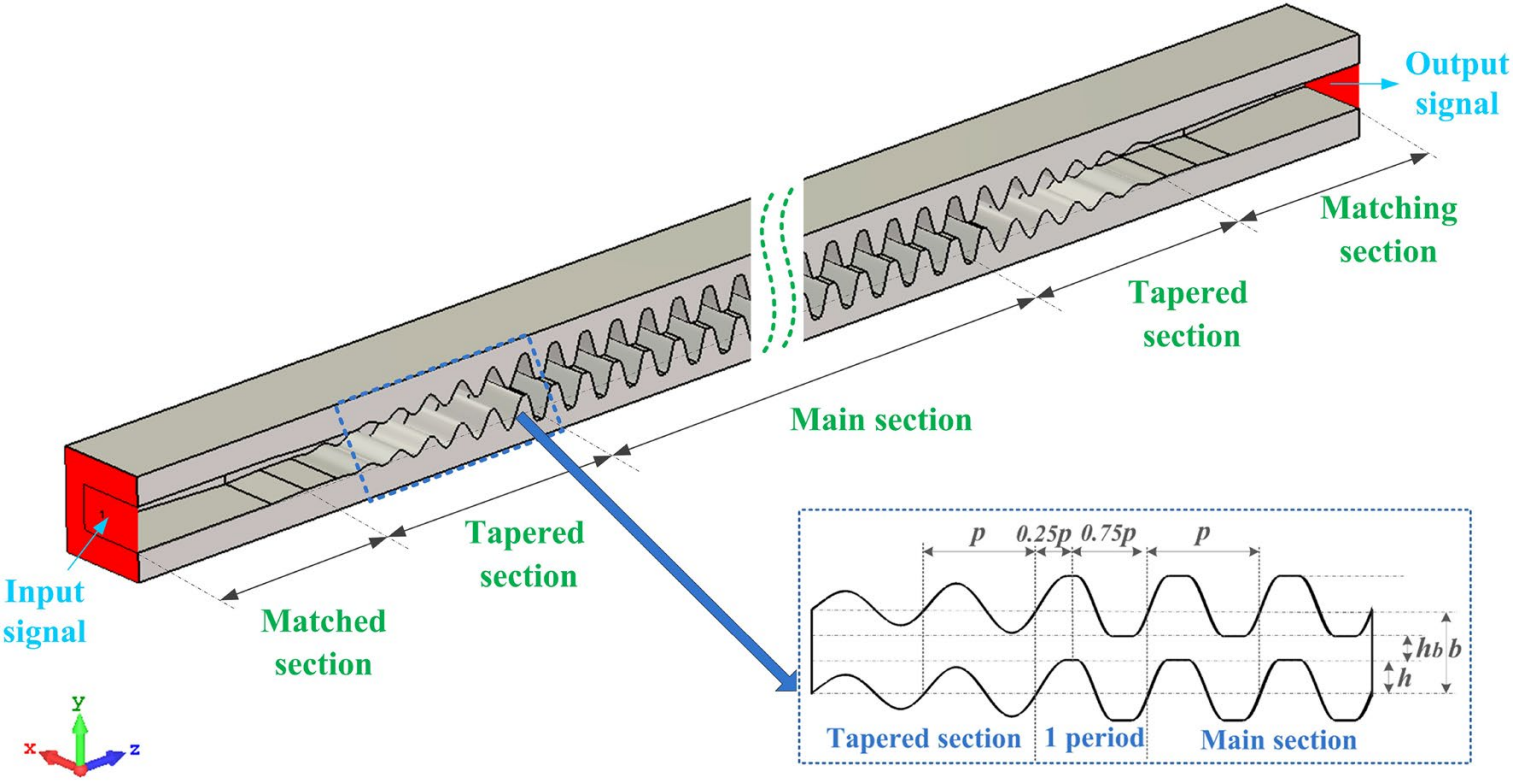
3D model of the PWSWG interaction circuit in CST MWS. The inset illustrates the design of the connect section.

As mentioned in the introduction, the transmission performance of the THz SWS is one of the most important factors for the THz TWT. A novel interaction circuit model based on the PWSWG SWS has been designed in CST-MWS^26^ (Fig. 8). The beam–wave interaction circuit model, comprises 50 periods of the PWSWG SWS, five periods of tapered section, and one period of connection section. The tapered sections are the SWG, and the oscillation amplitude *h* of the tapered section is gradually increased and decreased linearly to keep the perfect input and termination matching. In particular, the connect section, which comprises a quarter-period SWG and a three-quarter-period PWSWG connected at the peak position, was proposed to avoid the leap point as illustrated in the inset of Fig. 8. The tapered section and the WR4 standard waveguide were connected by inserting a gradient matching section. The total length of this model is 48 mm.

**Figure 9:**
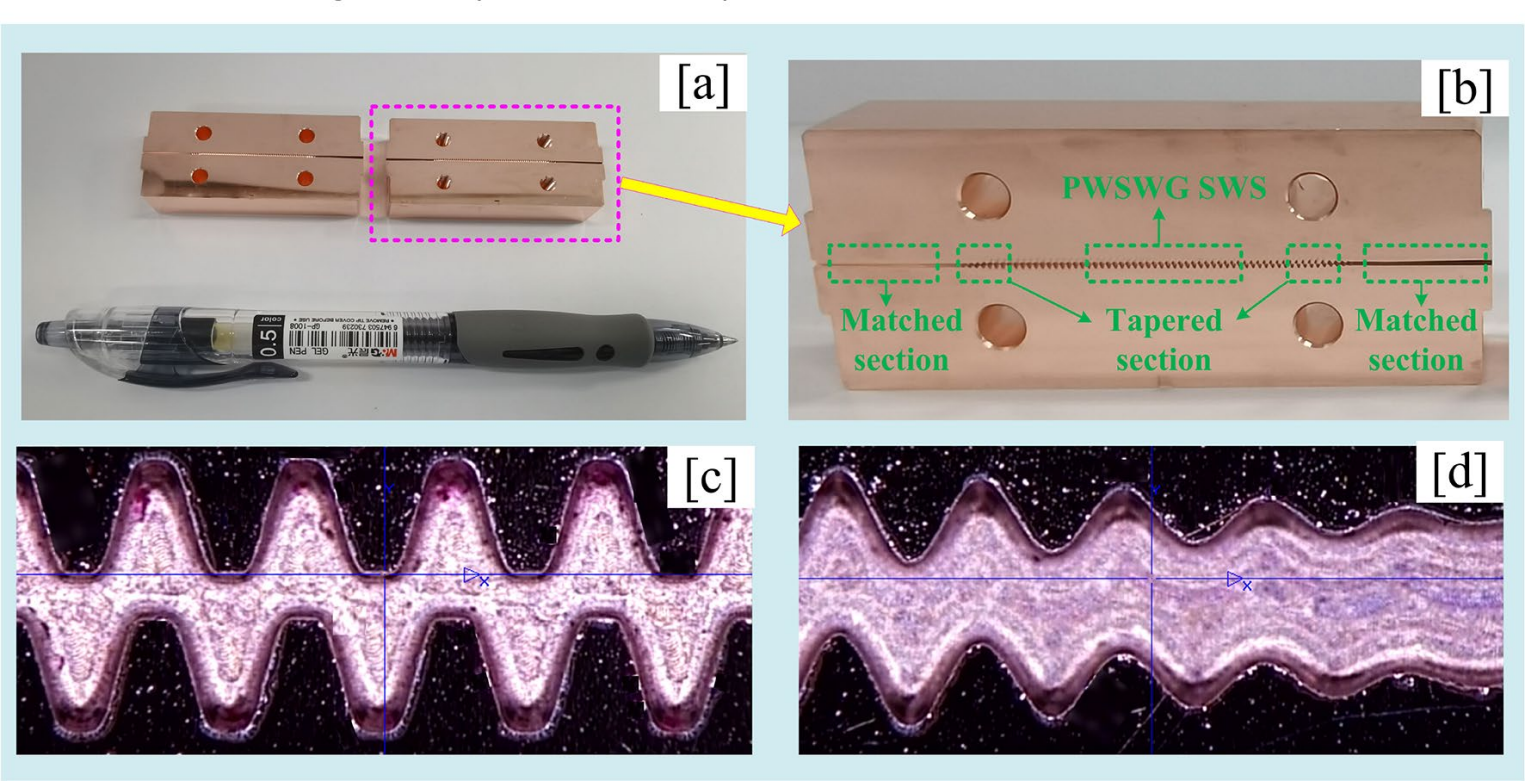
(**a**) Two parts of the fabricated circuit; (**b**) side view of the fabricated circuit; (**c**) the uniform section; and (**d**) the tapered section.

Next, the two parts of the interaction circuit model were fabricated via nano-CNC milling. The fabricated interaction circuit model, the contour of the uniform section, and the contour of the tapered section are illustrated in Fig. 9. As can be seen from Fig. 9, the boundary of the PWSWG SWS is very clear, and the actual machining accuracy is less than ± 3 µm.

**Figure 10:**
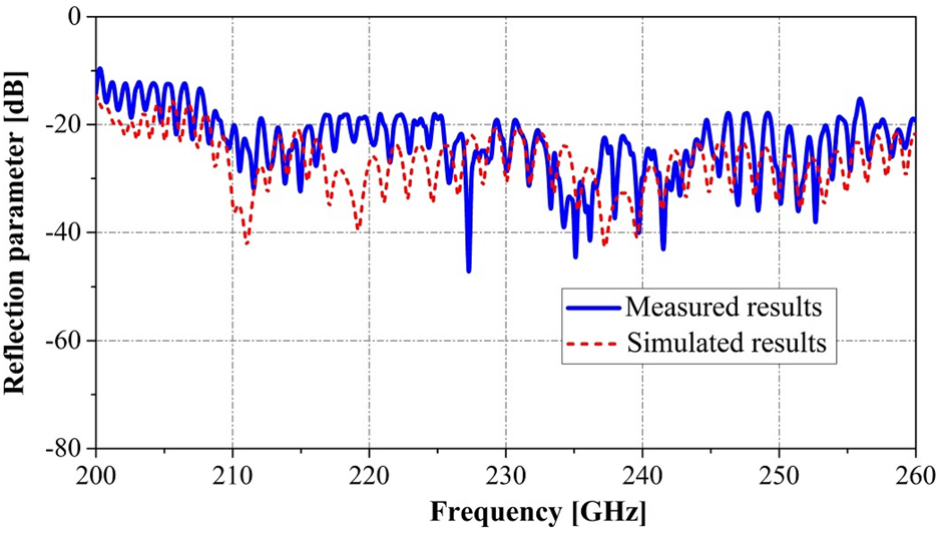
Comparison between measured results and simulated results of reflection parameters.

**Figure 11:**
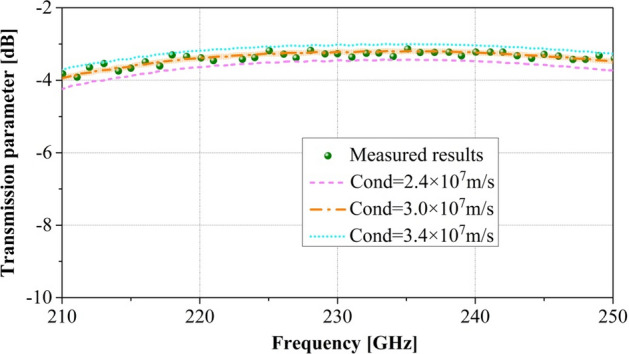
Comparison between measured results and simulated results of transmission parameters.

The transmission properties of the fabricated circuit were studied using the G-band frequency expansion module. As can be seen in Figs. 10 and 11, in the frequency range of 210–250 GHz, the experimental test results revealed that the reflection parameter and transmission parameter are less than − 18.1 dB and greater than − 3.91 dB, respectively. Thus, the PWSWG interaction circuit has good impedance matching and low loss characteristics. As shown in Fig. 10, the measurement results of reflection parameters are consistent with the simulated results. From the comparison in Fig. 11, it can be inferred that the equivalent conductivity for the PWSWG SWS in the frequency range of 210–250 GHz is between 2.4 × 10^7^ and 3.4 × 10^7^ S/m, and the corresponding actual conductivity at 220 GHz is approximately 3.0 × 10^7^ S/m. The beam–wave interaction performance of the PWSWG SWS can be predicted from the actual conductivity.

## Beam–wave interaction performance

**Figure 12:**
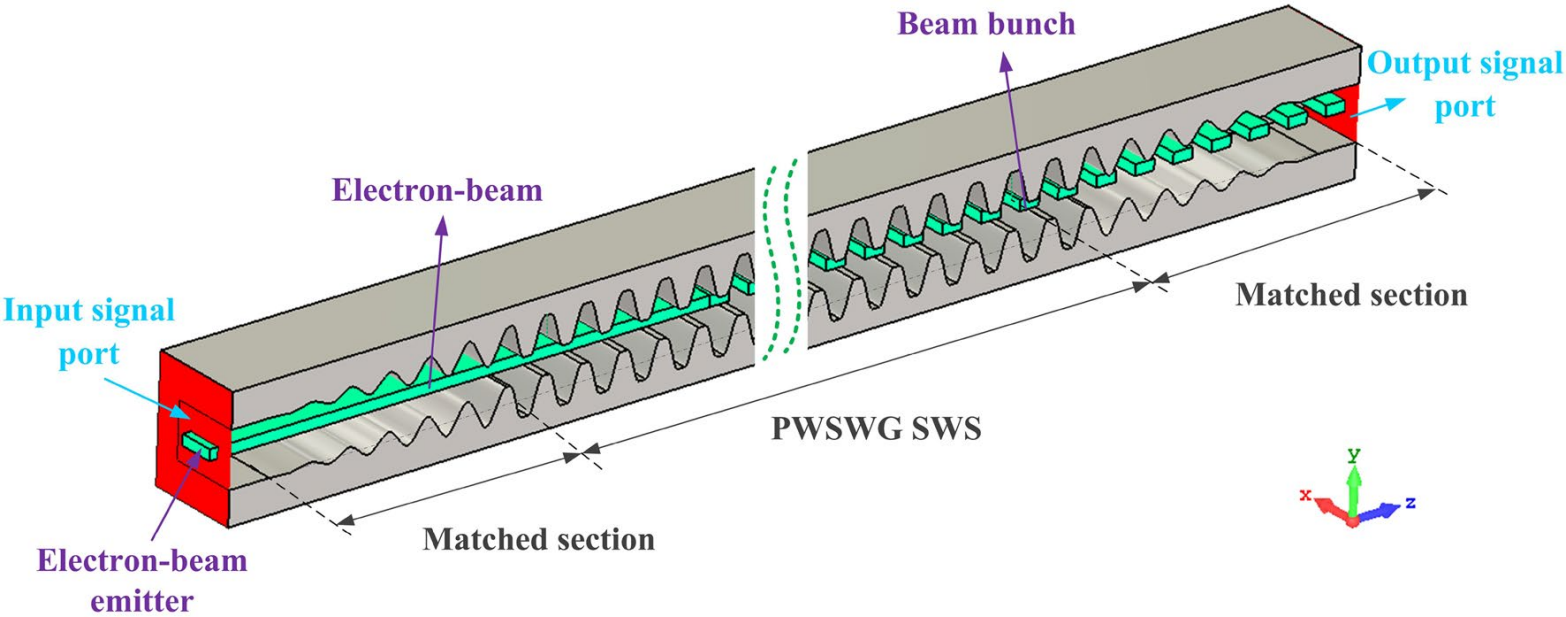
3D Particle-in-cell simulation model of the PWSWG TWT in CST PS.

The mechanism of beam–wave interaction in G-band PWSWG TWT can be simulated using 3D particle-in-cell (PIC) algorithms. The PIC algorithms in the CST PS are developed from the MAFIA (Solution of Maxwell’s equations by the finite-integration algorithm), widely used in the beam-wave interaction simulation of THz VEDs^27–30^. Based on the structural parameters obtained via optimization, we developed the PWSWG interaction circuit in the CST PS^31^ (Fig. 12). A sheet electron beam with an operating voltage of 20.9 kV and a current of 175 mA was passed through the middle of the tunnel. The cross-sectional area of the sheet electron beam was 400 × 100 µm^2^, and a uniform longitudinal magnetic of 1.5 T was applied here to confine the electron beam in the rectangular beam channel. Furthermore, a drive signal with an input power of 50 mW was applied to the input signal port.

In the PIC simulation, the electromagnetic wave propagates along the *z*-direction in the form of a guided wave, and the electron beam and electromagnetic wave exhibit identical propagation speeds. The electron beam and electromagnetic wave interact continuously in the SWS to realize the clustering of electron beams. During this process, most electrons decelerate, and a few accelerate, and the kinetic energy lost by the electrons is transformed into electromagnetic energy of the high-frequency signal. Thus, an amplified THz signal is obtained at the output port.

**Figure 13:**
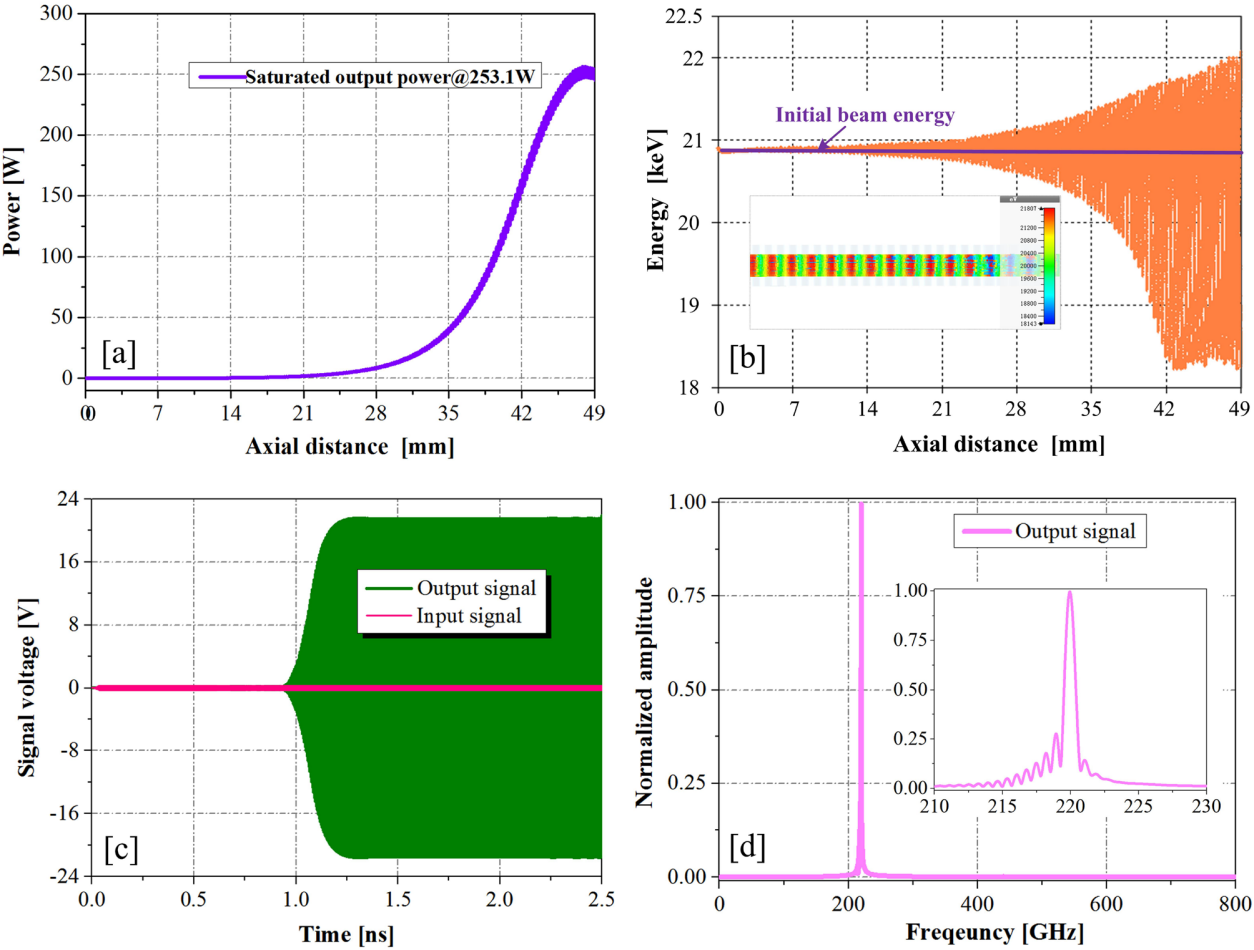
PIC simulation results (from CST) at 220 GHz. (**a**) Output power of the PWSWG interaction circuit as the function of the longitudinal distance. (**b**) The electron energy versus longitudinal distance. The inset shows the electron bunching at the end of interaction circuit. (**c**) Input and output signals. (**d**) Frequency spectrum of output signal.

**Figure 14:**
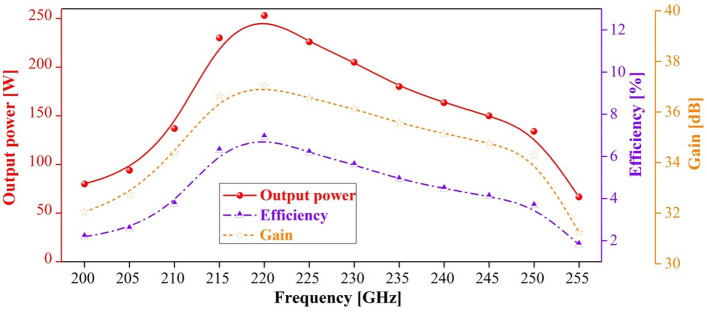
Output power, efficiency and gain of the PWSWG TWT versus frequency.

To achieve the saturation output power at the typical frequency of 220 GHz, the optimized value for the period numbers of the PWSWG interaction circuit was obtained as 100 by performing a large number of simulations. The typical PIC simulation results at 220 GHz were obtained via PIC simulation (Fig. 13). Figure 13a shows that the saturation output power can reach 251 W at the end of the PWSWG interaction circuit. From Fig. 13b, it can be seen that most electrons slowed down, and a few accelerated. For this reason, the majority of electrons lost their energy and gradually transformed into high-frequency electromagnetic field energy. As can be observed in the inset of Fig. 13b, the electron beam bunching occurred at the end of the beam–wave interaction circuit. This classical physical phenomenon usually occurs at the end of this TWT. It can be seen from Fig. 13c that the output signal reached stability after 1.25 ns of the beam–wave interaction. The drive signal amplitude increased from 0.31622 to 22.5 V, indicating a gain of 37.04 dB. In addition, the frequency spectrum was concentrated at 220 GHz and relatively pure, as evident from Fig. 13d. Because the termination was perfectly matched, the oscillation phenomenon was not observed. Nevertheless, an attenuator can be used to avoid the risk of oscillation in the practical device design^24^. To verify the amplitude–frequency response of the PWSWG TWT, the frequency point of the drive signal was swept with an input power of 50 mW. It can be seen in Fig. 14 that the 3-dB bandwidth exceeds 40 GHz with a maximum output power of 253.1 W and an interaction efficiency of 6.92%.

**Figure 15:**
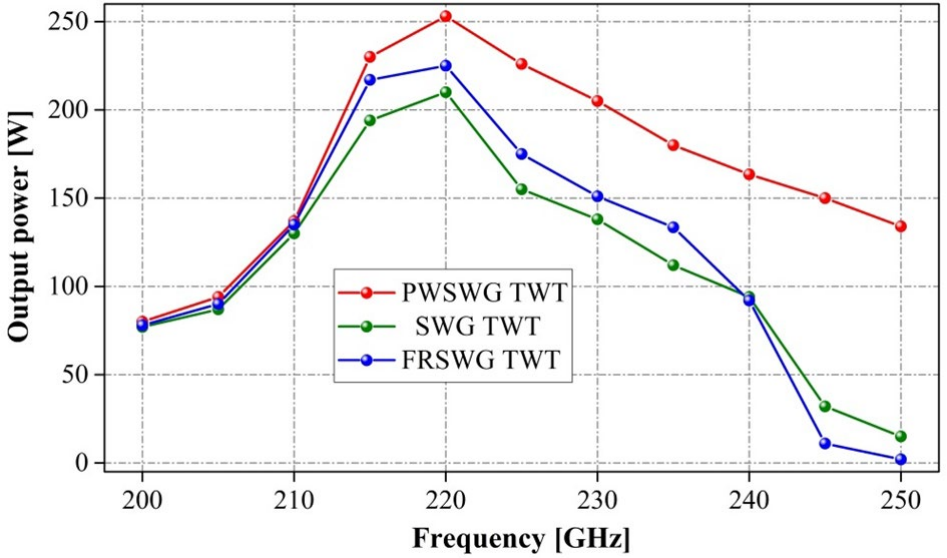
Comparison of output power of the PWSWG TWT, the SWG TWT and the FRSWG TWT.

Next, the SWG TWT and FRSWG TWT with almost the same dimensional parameters as described previously were developed in the CST PS. The synchronous voltage of these two TWTs was approximately 21.5 kV. To obtain the maximum output power at 220 GHz, the saturated period numbers of the SWG TWT and the FRSWG TWT were optimized as 120 and 105, respectively. The comparisons of the output power between the PWSWG TWT and the other two TWTs are shown in Fig. 15; the maximum output power of the PWSWG TWT at 220 GHz is approximately 15% and 20.5% greater than that of the SWG TWT and FRSWG TWT, respectively. Moreover, the PWSWG TWT possesses a wider bandwidth than the other two TWTs due to the flatter dispersion relation at high frequencies.

**Figure 16:**
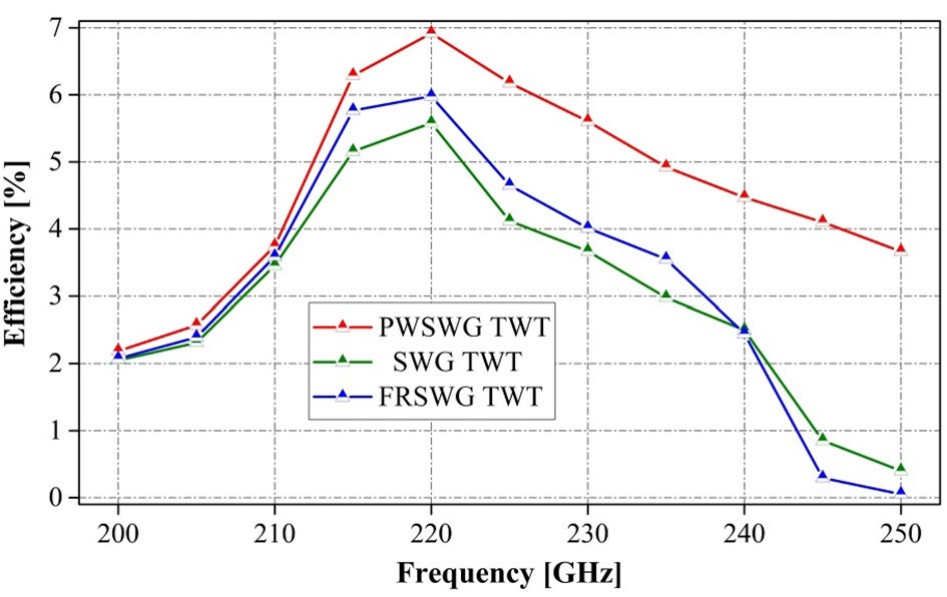
Comparison of output power of the PWSWG TWT, the SWG TWT and the FRSWG TWT.

**Figure 17:**
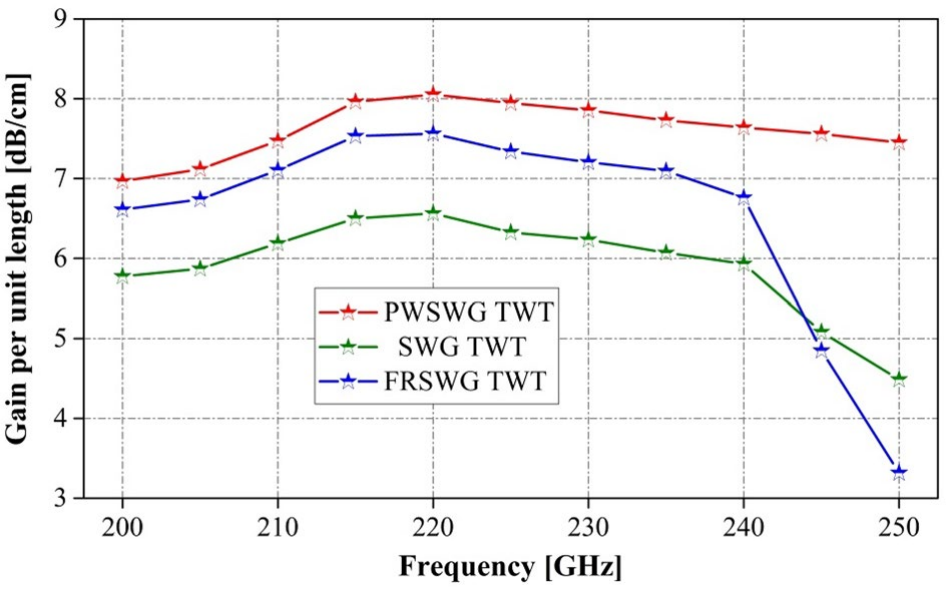
Comparison of gains per unit length of the PWSWG TWT, the SWG TWT and the FRSWG TWT.

The interaction efficiency of the PWSWG TWT was compared with that of the SWG TWT and FRSWG TWT (Fig. 16). It was found that the interaction efficiency of the PWSWG TWT is higher than that of the other two TWTs in the frequency range of 200–250 GHz. In addition, the gains per unit length of the three TWTs were compared to analyze the amplifying performance of the SWS. As shown in Fig. 17, the gain per unit length of the PWSWG TWT exceeds 7.47 dB/cm in the frequency range of 210–250 GHz, thereby indicating that the gains per unit length of the PWSWG TWT are much higher than those of the other two TWTs over the entire operating band.

## Summary

A PWSWG SWS that possesses the advantages of large interaction impedance and excellent electromagnetic transmission performance was proposed in this paper. The simulation results revealed that the interaction impedance of the PWSWG SWS is higher than that of conventional SWG SWSs because of the improvement in the longitudinal electrical field amplitude. In addition, the excellent electromagnetic transmission properties of the PWSWG SWS were verified via simulation and cold tests. Moreover, the beam–wave interaction results showed that the PWSWG TWT has higher radiated power, higher interaction efficiency, and superior amplifying performance than the conventional SWG TWTs. Therefore, this PWSWG should be considered a promising SWS for wide-band high-power THz radiation sources.

## Data Availability

The datasets used and/or analysed during the current study available from the corresponding author on reasonable request.
